# Engineering of Effector Domains for Targeted DNA Methylation with Reduced Off-Target Effects

**DOI:** 10.3390/ijms21020502

**Published:** 2020-01-13

**Authors:** Daniel Hofacker, Julian Broche, Laura Laistner, Sabrina Adam, Pavel Bashtrykov, Albert Jeltsch

**Affiliations:** Institute of Biochemistry and Technical Biochemistry, Department of Biochemistry, Stuttgart University, Allmandring 31, 70569 Stuttgart, Germany; Daniel.Hofacker@hotmail.de (D.H.); julian.broche@ibc.uni-stuttgart.de (J.B.); laura.laistner@web.de (L.L.); sabrina.adam@ibtb.uni-stuttgart.de (S.A.)

**Keywords:** epigenome editing, targeted DNA methylation, dCas9, SunTag, DNMT3A-DNMT3L

## Abstract

Epigenome editing is a promising technology, potentially allowing the stable reprogramming of gene expression profiles without alteration of the DNA sequence. Targeted DNA methylation has been successfully documented by many groups for silencing selected genes, but recent publications have raised concerns regarding its specificity. In the current work, we developed new EpiEditors for programmable DNA methylation in cells with a high efficiency and improved specificity. First, we demonstrated that the catalytically deactivated Cas9 protein (dCas9)-SunTag scaffold, which has been used earlier for signal amplification, can be combined with the DNMT3A-DNMT3L single-chain effector domain, allowing for a strong methylation at the target genomic locus. We demonstrated that off-target activity of this system is mainly due to untargeted freely diffusing DNMT3A-DNMT3L subunits. Therefore, we generated several DNMT3A-DNMT3L variants containing mutations in the DNMT3A part, which reduced their endogenous DNA binding. We analyzed the genome-wide DNA methylation of selected variants and confirmed a striking reduction of untargeted methylation, most pronounced for the R887E mutant. For all potential applications of targeted DNA methylation, the efficiency and specificity of the treatment are the key factors. By developing highly active targeted methylation systems with strongly improved specificity, our work contributes to future applications of this approach.

## 1. Introduction

**Figure 1:**
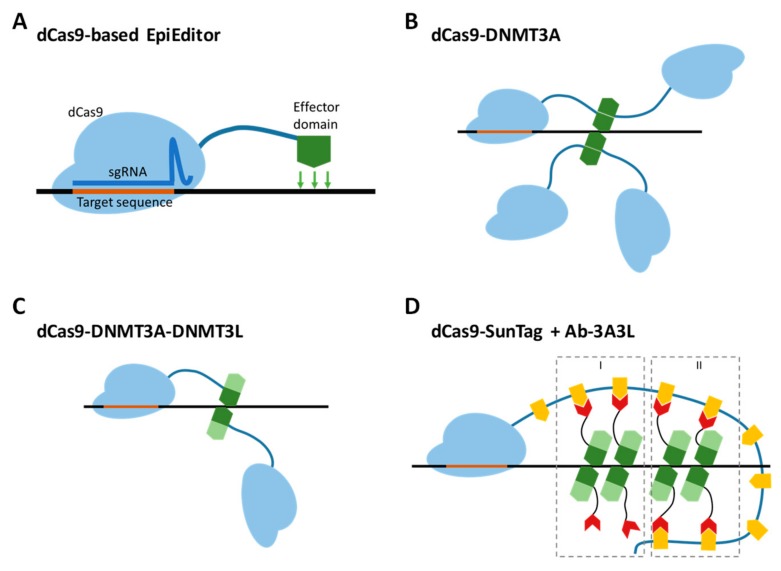
EpiEditors for targeted DNA methylation. (**A**) General design of an EpiEditor containing a chromatin-modifying enzyme (effector module, green) fused to a catalytically deactivated Cas9 protein (dCas9, light blue) in complex with a single guide RNA (sgRNA, dark blue) complementary to the target genomic locus. (**B**) The direct fusion protein of dCas9 and the C-terminal domain of DNMT3A form a tetramer at a target site to produce a catalytically active complex. (**C**) The dCas9 direct fusion protein with the fused C-terminal domains of DNMT3A (dark green) and DNMT3L (light green) form an active complex by dimerization. (**D**) Co-expression of two chimeric proteins, dCas9-SunTag and Ab-3A3L (containing the fused C-terminal domains of DNMT3A and DNMT3L, dark and light green), leads to the formation of active complexes by dimerization of recruited and free Ab-3A3L (mechanism I) or only by interaction of two recruited Ab-3A3L dimers (mechanism II). The orange shapes represent the GCN4 peptide sequence, the red shapes represent the scFv antibody binding to the GCN4 peptide.

The targeted alteration of gene expression is a widely used approach in basic research as well as in the development of new therapeutic strategies. For decades, the toolbox available for this ambition was limited to two main techniques, namely gene knockdown and knockout, which either reduce or completely inactivate gene function [1,2]. Recently, a new technology, called epigenome editing, emerged and allowed programmable silencing as well as activation of gene expression [3,4]. Tuning of gene expression can be achieved by modulation of epigenomic signals, which regulate transcription at gene control elements, mainly promoters. This fundamental difference in the mechanism of action from the predecessors gives rise to three key advantages: established expression profiles can be stably maintained by cellular epigenetic machinery; they are reversible; and the method does not change the genetic information. The key instruments for epigenome editing are the so-called EpiEditors, artificial proteins constructed from two functional units, one of which is responsible for the delivery of the construct to the targeted genomic region and the other for the local modification of the chromatin state to establish the desired properties. Currently, EpiEditors are mainly created using the catalytically inactive Cas9 protein (dCas9) fused to chromatin-modifying enzymes [5]. To produce a functional DNA binding unit, dCas9 needs to form a complex with a single guide RNA (sgRNA), which is designed to contain a sequence complementary to the targeted genomic locus next to a protospacer adjacent motif site (Figure 1A) [6,7,8].

Several flavors of EpiEditors were developed and they demonstrated their ability to activate or silence gene expression by removing or depositing epigenetic marks [3]. DNA methylation of CpG islands in gene promoters is a regular mechanism for gene inactivation in mammalian cells [9] and targeted DNA methylation was an early example successfully demonstrating the feasibility of epigenome editing [10]. Targeted DNA methylation and the following gene repression were achieved using EpiEditors containing zinc finger proteins fused to the catalytic domain of DNA methyltransferase DNMT3A [10,11,12,13,14].

Two important characteristics of epigenome editing, attracting a lot of attention, are its on-target activity and specificity (i.e., the methylation activity at on-target versus off-target sites). In vitro studies demonstrate that DNMT3L stimulates the activity of DNMT3A [15,16]. Inspired by this, an improved EpiEditor using a DNMT3A-DNMT3L single-chain fusion protein as an effector domain was developed [17]. It resulted in increased DNA methylation activity and was applied in several studies for gene silencing [17,18,19,20]. Another advancement was the introduction of dCas9 as a programmable DNA binding domain, which was used in several projects aiming for targeted DNA methylation [19,20,21,22,23,24,25,26,27]. Recently, the epigenome editing toolbox was developed further due to the adaptation of a dCas9-SunTag platform [28] allowing the recruitment of up to 24 effector proteins to one target locus. This system consists of dCas9 fused to an array of peptide sequences, which are derived from the GCN4 protein and recognized by a single-chain antibody (scFv-GCN4). Co-expression of dCas9-SunTag and scFv-GCN4 fused to chromatin-modifying enzymes results in the recruitment of multiple effector domains to the target locus and may potentially increase on-target editing efficiency, which was demonstrated with the TET1 catalytic domain used for DNA demethylation [29,30] as well as the DNMT3A catalytic domain used for DNA methylation [24,26].

In parallel, concerns have been raised regarding the specificity of targeted DNA methylation during the last years, because several studies documented off-target methylation at multiple loci using genome-wide analysis [25,26,27]. It was reported that the specificity of the dCas9-SunTag system is higher in comparison to the direct fusion constructs [24,26]. Nevertheless, to achieve an optimum specificity, a thorough titration of the scFv-DNMT3A construct was necessary, which led to a lowering of the on-target activity. In the current work, we aimed to further develop EpiEditors for targeted DNA methylation with increased on-target activity and reduced off-target effects. To achieve this, we combined the dCas9-SunTag system with an effector protein comprising the fused C-terminal domains of DNMT3A and DNMT3L, which demonstrated strong methylation of the target locus. Further, we designed several DNMT3A mutants to reduce the endogenous DNA binding affinity of the DNMT3A subunit, which led to a drastic reduction of global off-target DNA methylation, but only a mild loss of on-target methylation, resulting in strongly improved specificities of epigenome editing.

## 2. Results

### 2.1. Comparison of the Efficiency and Specificity of the dCas9-DNMT3A-DNMT3L Direct Fusion with the dCas9-SunTag System

We started the project with a careful selection of the platform for targeted DNA methylation. As an editing module, there are two options available so far, which are a catalytic domain of DNMT3A or its fusion with the C-terminal part of DNMT3L. As published earlier, the C-terminal part of DNMT3L stimulates the activity of DNMT3A in vitro and in vivo [16,17]. Apart from that, the smallest catalytically-active unit of DNMT3A is a tetramer consisting of either two DNMT3A homodimers or two DNMT3A-DNMT3L heterodimers [31,32,33]. Due to this, an EpiEditor containing a DNMT3A-DNMT3L fusion is structurally more preferable, since it already provides one assembled heterodimer. Thus, in the case of dCas9-DNMT3A, recruitment of four molecules is required to generate one catalytically active tetramer (Figure 1B), while dCas9 fused to DNMT3A-DNMT3L needs only to dimerize to form an active complex (Figure 1C). The dCas9-SunTag provides a scaffold for the recruitment of several chimeric scFv-GCN4-DNMT3A-DNMT3L (Ab-3A3L) proteins with two potential mechanisms of assembly (Figure 1D). In the first mechanism, active heterotetramers can be formed by one DNMT3A-DNMT3L dimer bound to the SunTag with a second unbound dimer by direct DNMT3A-DNMT3A interface interaction. In the second one, two SunTag bound dimers can interact with each other. Both mechanisms allow the recruitment of several active tetramers by a single sgRNA-dCas9 complex and theoretically higher activity in comparison to the Ab-3A system published earlier [24,26]. Thus, we decided to compare the dCas9-DNMT3A-DNMT3L (dC) and dCas9-10XSunTag/Ab-3A3L (dCS) systems.

**Figure 2:**
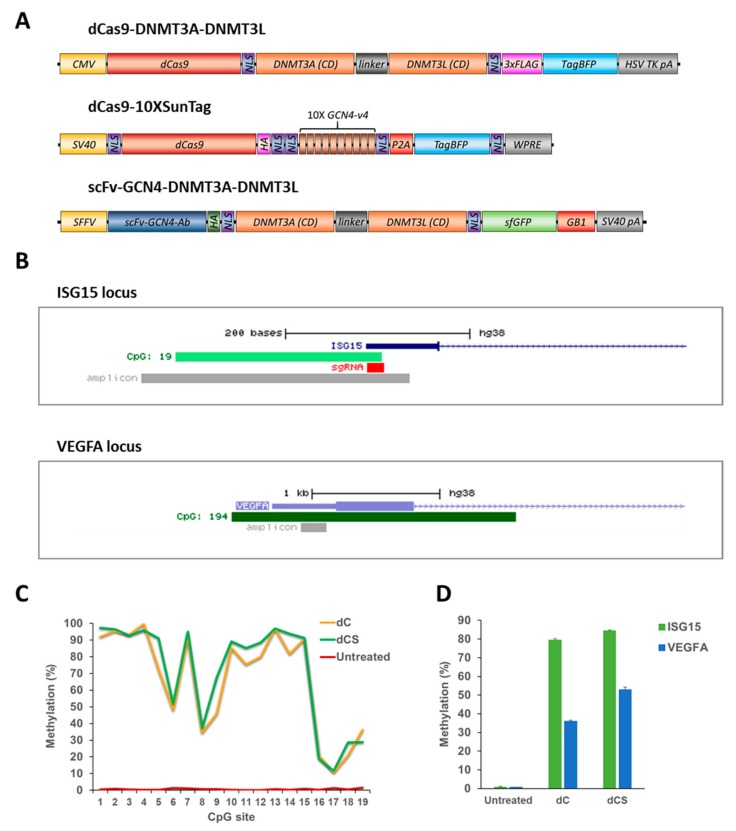
Comparison of the efficiency and specificity of the dCas9-DNMT3A-DNMT3L direct fusion (dC) and dCas9-10XSunTag/Ab-3A3L (dCS) systems. (**A**) Schematic drawing of the direct and SunTag based systems used in the study. (**B**) UCSC genome browser views of the *ISG15* (chr1:948671-948894, hg19) and *VEGFA* (chr6:43737633-43739852, hg19) promoter regions showing the localization of CpG islands (in light and dark green), amplicons used in targeted bisulfite sequencing (bis-seq), and sgRNA binding site within the *ISG15* CGI. (**C**) DNA methylation of individual CpG sites (on the x-axis) at the *ISG15* region determined by targeted bis-seq in untreated HEK293 cells and after treatment with the dC or dCS system using Ab-3A3L and the ISG15 sgRNA. Data from a single representative experiment are shown. (**D**) Average DNA methylation at the target (*ISG15*) and off-target (*VEGFA*) regions were analyzed by targeted bis-seq of HEK293 in untreated cells and after targeted methylation with the dC or dCS systems. The figure shows the means of these average methylation levels based on two independent experiments and the standard deviations of the means. See also Figure S2.

The DNA methylation efficiency and specificity of the dC and dCS systems (Figure 2A) were investigated at two loci, namely a CpG island (CGI) in the promoter of the *ISG15* gene targeted by an ISG15 sgRNA and a CGI in the *VEGFA* gene promoter as an off-target region (Figure 2B), which both are unmethylated in the HEK293 cell line (Figure 2C,D). According to our previous experience, the *VEGFA* CGI is readily methylated by EpiEditors, so it is a sensitive genomic region, which is suitable for the measurement of the off-target activity of epigenome editing in screening experiments. The standardized workflow used in this experiment as well as in all others was as follows: HEK293 cells were transiently co-transfected with a cocktail of plasmids including the expression vector for the ISG15 sgRNA and the vectors to express Ab-3A3L and dC or dCS. All vectors contained fluorescent markers: the sgRNA expression plasmid carries DsRed, dC and dCS contain tagBFP, and Ab-3A3L sfGFP. Three days later, cells were collected and sorted by flow cytometry to assure that only cells carrying all components were included in the downstream analysis. DNA methylation at the *ISG15* and *VEGFA* CGIs was analyzed by targeted bisulfite sequencing (bis-seq). Controls revealed that the bisulfite conversion rate was >99.5% in all analyzed samples (Figure S1).

Initial results demonstrated that both EpiEditors were able to methylate the targeted *ISG15* locus very efficiently (Figure 2C,D). Methylation levels of individual CpG sites varied but reached more than 95% for selected ones. Since the sgRNA target sequence was covered by the amplicon used for the bis-seq analysis, CpG sites 16–19 were blocked by the dCas9-sgRNA complex. Therefore, they were not accessible for DNMT3A and consequently showed low methylation levels. The average DNA methylation level in the analyzed region (CpGs 1 to 15) was about 79% and 84% for dC and dCS, correspondingly (Figure 2D).

Next, we analyzed the methylation of the *VEGFA* CGI off-target region and found that it was methylated to 36% and 53% by the dC and dCS, respectively (Figure 2D). Thus, off-target methylation was high for both constructs and the dCas9-10XSunTag vector showed an even higher off-target methylation. Hence, different from other published papers, we could not observe a higher specificity of the SunTag-based vector. This difference could be due to differences in the experimental conditions and the expression levels of effector domains and/or the overall higher activity of DNMT3A-DNMT3L. This result indicates that the specificity of targeted methylation depends on the exact experimental conditions and the experimental outcome of targeted DNA methylation may, therefore, fluctuate depending on the cell line and the expression levels of the EpiEditors in each particular experiment. Thus, there is a need for a more robust and specific system for targeted DNA methylation.

### 2.2. Rationally Designed Mutations in DNMT3A Decrease Off-Target Methylation

Off-target methylation of the EpiEditors may originate from two sources. Firstly, dCas9 can bind sequences partially matching the target DNA, especially if the mismatch(es) are within the 5′ end of the guide RNA [34]. This will recruit DNA methyltransferase activity to undesired loci. To test this hypothesis, we analyzed potential off-targets for our sgRNA using a web-based tool (http://www.rgenome.net/cas-offinder/) [35]. However, it identified only 14 regions with three mismatches and 94 regions with four mismatches (Table S1) to the used ISG15 sgRNA and the closest match was about 3 million base pairs away from the analyzed region in the *VEGFA* CGI. Based on this analysis, it is unlikely that the methylation of this region appears due to off-target DNA binding of dCas9. Secondly, DNMT3A can bind DNA independently of dCas9 and this can lead to methylation of accessible DNA in open chromatin. This effect will be more likely in cells with a high expression of the EpiEditors, where an excess of the DNA methyltransferase freely diffuses and is not recruited to the target locus. Based on this, we hypothesized that weakening of the DNMT3A-DNA interaction may reduce DNA methylation selectively at undesired loci, because recruitment to the target locus is mediated by dCas9 and less dependent on the endogenous DNA binding capabilities of DNMT3A.

**Figure 3:**
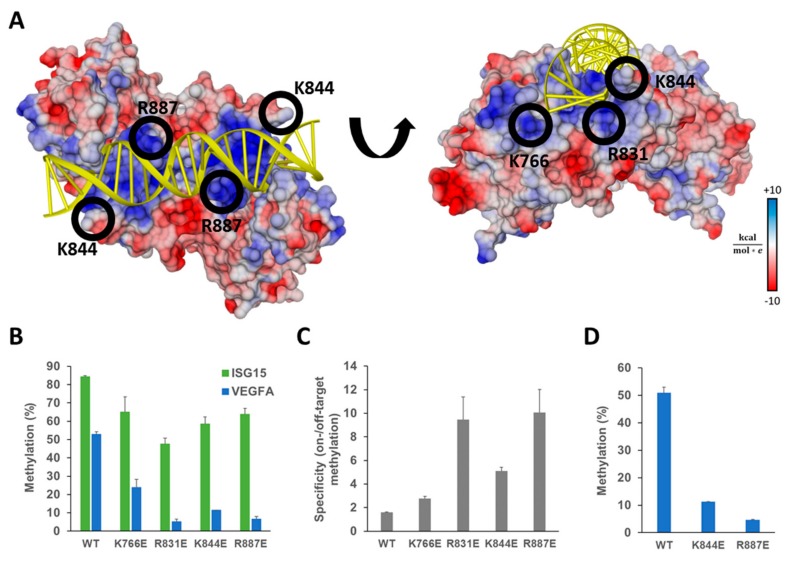
Ab-3A3L containing DNMT3A mutants with reduced DNA binding shows lower off-target methylation but retains most of the on-target activity. (**A**) Crystal structure of the DNMT3A-DNA complex (pdb 5YX2, [36]) showing the DNA (yellow) bound to the central dimeric DNMT3A catalytic domain colored according to the surface charge (blue positive, red negative). Positively charged residues on the protein surface (blue patches) interact with the negatively charged DNA phosphodiester backbone. Amino acids K766, R831, K844, and R887 were selected for mutagenesis and replaced by glutamic acid to reduce the strength of the protein–DNA interaction. (**B**) DNA methylation of the target (*ISG15*) and off-target (*VEGFA*) regions analyzed by targeted bis-seq in HEK293 cells using the dCS system with ISG15 sgRNA and DNMT3A wild type and mutants in Ab-3A3L. Results for the wild type DNMT3A are taken from Figure 2D and shown for comparison. Data present the mean of two independent experiments and error bars show the standard deviation. *p*-Values for comparison of the off-target activity of the mutants with wild type were 9.36 × 10^−3^ (K766E), 4.19 × 10^−4^ (R831E), 2.69 × 10^−4^ (K844E), and 5.05 × 10^−4^ (R887E) (one-sided t-test assuming unequal variance based on the experimental repetitions). (**C**) The specificity of the EpiEditors calculated as a ratio of the on- versus off-target DNA methylation based on the data of (B). (**D**) Off-target methylation analyzed at the *VEGFA* CGI for Ab-3A3L containing wild type and mutated DNMT3A co-transfected with dCS and the scrambled sgRNA coding vector. See also Figure S2.

To test this hypothesis, we analyzed the DNMT3A-DNA crystal structure (pdb 5YX2, [36]) and identified amino acids involved in an interaction with the DNA backbone (Figure 3A). Several lysine and arginine residues formed a positively charged patch on the surface of the protein, from which we selected K766, R831, K844, and R887 for future experiments. To reduce DNA binding strongly, charge reversal mutations were introduced and the selected lysine or arginine residues were exchanged by glutamic acid. Previous work already demonstrated that mutagenesis of R831, K844, and R887 weakens DNA binding, but it also decreases the catalytic activity of DNMT3A in vitro [37,38], which makes it likely that it will decrease on-target DNA methylation in vivo as well. To minimize the possible negative effects of reduced catalytic activity, we decided to test Ab-3A3L containing the DNMT3A mutants in the dCas9-SunTag system, since it can recruit up to ten effector domains, which may compensate for the reduction of DNA methylation activity in comparison to the dCas9-3A3L direct fusion.

The four single amino acid mutants K766E, R831E, K844E, and R887E were generated in the Ab-3A3L vector using site-directed mutagenesis. The obtained variants were individually tested in HEK293 cells co-transfected with dCS and the same guide RNA targeting the *ISG15* promoter CGI as before. DNA methylation was analyzed by targeted bis-seq and it demonstrated that all mutants are functional in vivo at the target site (Figure 3B), though they showed minor differences in activity levels. The K766E and R887E mutants introduced the highest DNA methylation at the analyzed region with 65% and 64% methylated CpG sites, and K844E and R831E showed 58% and 48% of methylation, respectively. When compared with the wild type Ab-3A3L, we observed 56%, 69%, 76%, and 77% residual activity in the case of R831E, K844E, R887E, and K766E, respectively.

Analysis of the DNA methylation at the *VEGFA* locus revealed a strong and highly significant reduction of the off-target DNA methylation introduced by the mutants in comparison to the wild type (Figure 3B). The K766E and K844E mutants showed 55% and 78% reduction and the best results were achieved with the R887E and R831E variants showing 88% and 90% less methylation at the off-target site. To compare the constructs with respect to an optimum combination of high on-target activity and low off-target effects, we calculated their specificity as a ratio between the on- and off-target DNA methylation. The obtained results showed that the system comprising dCS and wild type Ab-3A3L had a specificity factor of only 1.6, indicating a very low preference for the target site. In contrast, the two best mutants, R887E and R831E, reached specificity ratios of 10.1 and 9.4, respectively (Figure 3C). The two other mutants, K766E and K844E, showed a moderate, but still detectable, increase in specificity to ratios of 2.8 and 5.1. Thus, our design was successful and resulted in the generation of EpiEditors with increased specificity.

### 2.3. Additional Off-Target Methylation Experiments

Next, we performed an additional experiment aiming to confirm that the off-target methylation is a result of the DNA methylation at accessible regions by the Ab-3A3L subunit and independent of dCas9 recruitment. To this end, cells were co-transfected with vectors expressing dCS, the wild type Ab-3A3L or mutants of it, and a scrambled sgRNA designed to have minimum similarity with the human genome [39]. Since we aimed to achieve an ideal compromise between high activity and high specificity of our EpiEditors, the K844E and R887E mutants were used, but the R831E mutant, which showed the weakest activity at the target locus, was not included in this follow-up experiment. As shown in Figure 3D, the wild type, K884E and R887E Ab-3A3L variants methylated the *VEGFA* locus in this experiment as well. The DNA methylation levels were very similar to the ones obtained in the experiment with the ISG15 sgRNA (Figure 3B) indicating that off-target activity indeed reflects the accessibility of the particular locus for the freely diffusing Ab-3A3L proteins.

So far, the *VEGFA* locus has been used as a marker for off-target methylation in screening experiments and demonstrated an improved specificity of all mutants. However, for a global picture we analyzed genome-wide DNA off-target methylation using the MBD-seq assay for selected samples. MBD-seq is established in many publications as a reliable and cost-efficient method for genome-wide DNA methylation analysis at medium resolution [40]. HEK293 cells were treated with dCS, the ISG15 sgRNA, and the Ab-3A3L containing wild type, R887E or K844E DNMT3A. To compare global methylation levels, genomic DNA was isolated from treated and untreated cells and sonicated. Then, methylated DNA fragments were enriched by MBD2-pulldown and after library preparation, sequenced on Illumina HiSeq3000.

**Figure 4:**
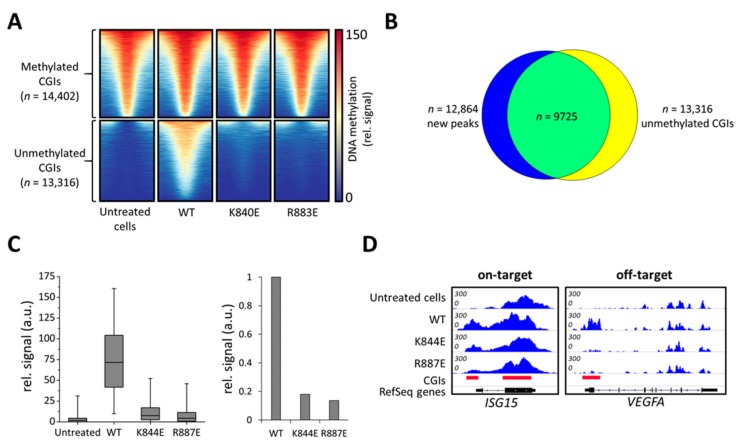
Genome-wide analysis of off-target DNA methylation in cells treated with dCS, Ab-3A3L with wild type or mutant DNMT3A parts, and ISG15 sgRNA. (**A**) Heatmap showing DNA methylation centered around CGIs ± 1 kb. Untreated cells contain methylated and unmethylated CGIs, which are separated into two groups shown in the upper and lower panels. The wild type DNMT3A (WT) construct caused a very strong de novo methylation in previously unmethylated CGIs, whereas K844E and R887E showed drastically lower methylation. (**B**) Intersection of new DNA methylation peaks generated by the wild type DNMT3A (WT) and the group of unmethylated CGIs taken from (A). (**C**) Boxplot showing a quantification of MBD-seq data sets using the group of unmethylated CGIs from (A) (line = median, box = 25th and 75th percentile, whiskers = 5th and 95th percentile). The bar diagram shows average signals in unmethylated CGIs normalized to wild type (WT). (**D**) Integrative Genomics Viewer (IGV) browser views of MBD-seq data displayed for the *ISG15* (chr1:948,441-950,302, hg19) and *VEGFA* (chr6:43,736,149-43,754,992, hg19) regions. For quantification and additional off-target loci, see Figures S4 and S5.

In untreated cells, the 27,718 CGIs in the human genome were subdivided based on their methylation signal into two groups, unmethylated (*n* = 13,316) and methylated (*n* = 14,402) (Figure 4A). Strikingly, a strong increase in the MBD2-pulldown DNA methylation signal was observed in cells transfected with the wild type construct. Peak calling identified 12,864 new MBD-seq peaks compared to untreated cells, out of which 10,921 were located in CGIs (Figure S3) and 9725 peaks overlapped with originally unmethylated CGIs (Figure 4B). Therefore, we focused our further analysis on unmethylated CGIs. In contrast to wild type DNMT3A, both tested mutants, K844E and R887E, showed only a slight increase in DNA methylation levels in untargeted CGIs (Figure 4A,C). For a quantitative comparison, the MBD-seq signals in unmethylated CGIs were averaged showing a 6-fold reduction in methylation for K844E and even a 7.8-fold reduction for R883E compared to the wild type (Figure 4C). Analysis of the targeted *ISG15* locus confirms that both mutants were able to introduce a methylation signal at the target site despite their strongly reduced activity at off-target loci like the *VEGFA* promoter (Figure 4D, Figure S4). These effects are even more pronounced in other off-target loci like *ACTB* or *GAPDH* (Figure S5). Quantitative analysis of the MBD-seq peak areas confirmed the strong improvement of K844E and R887E in editing specificity (Figure S4). Thus, the DNMT3A variants designed here showed a high specificity of targeted DNA methylation due to strongly reduced off-target methylation.

### 2.4. 24XSunTag Does Not Increase On-Target DNA Methylation

**Figure 5:**
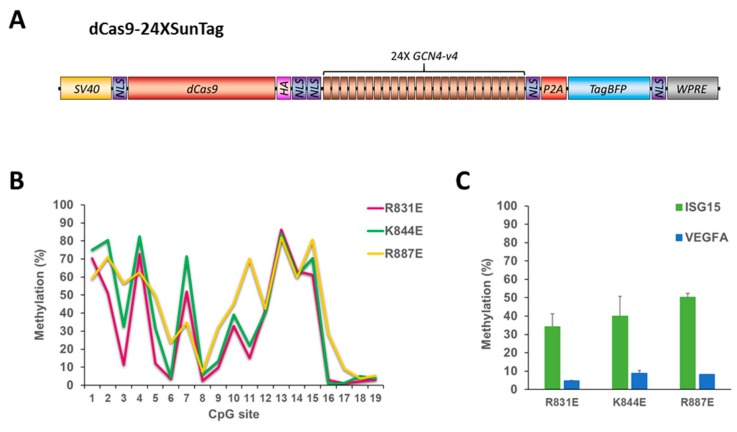
The dCas9-24XSunTag does not increase on-target DNA methylation. (**A**) Scheme of the dCas9-24XSunTag (dCS24) vector used in this study. (**B**) DNA methylation of individual CpG sites (on the x-axis) of the *ISG15* region determined by targeted bis-seq after treatment of HEK293 cells with the dCS24, ISG15 sgRNA, and Ab-3A3L with the R831E, K844E, and R887E mutations in the DNMT3A part. Data from a single representative experiment are shown. (**C**) A mean methylation of the target (*ISG15*) and the off-target (*VEGFA*) regions analyzed by targeted bis-seq of HEK293 based on two independent experiments. Error bars show standard deviations. Note that higher methylation levels were observed with the dCas9-10XSunTag with the same Ab-3A3L mutants (Figure 3B). See also Figure S2.

Since all generated DNMT3A variants showed reduced on-target activity, we thought that it may be possible to improve on-target methylation by recruiting even more effector domains. To this end, we used the dCS vector fused to 24 SunTag repeats (dCS24) in combination with the Ab-3A3L containing the DNMT3A mutations R831E, K844E or R887E and the ISG15 sgRNA (Figure 5). While we observed methylation levels of up to 80% at some CpG sites in the *ISG15* target site with dCS24, surprisingly the overall DNA methylation was lower in comparison to the dCS with all three mutants. In contrast, off-target methylation stayed as low as it was demonstrated with dCS, confirming that it is a function of DNA methyltransferase and not related to off-target binding of the dCas9 protein. Thus, the use of 24X SunTag did not increase on-target DNA methylation in comparison to the 10X SunTag array.

## 3. Discussion

The efficiency of targeted DNA methylation depends on the effector domains used to generate an EpiEditor. It was previously shown, that the DNMT3A-DNMT3L fusion protein is more active both in vitro and in vivo than an isolated DNMT3A catalytic domain [17,20]. Using dCas9-SunTag proteins for targeting can even further increase on-target activity, because they are recruiting several effector domains to a target genomic locus, which was demonstrated with the DNMT3A catalytic domain [24,26,29] as well as with the TET1 catalytic domain used for DNA demethylation [29,30]. In the current study and for the first time, we combined the scFv-GCN4-DNMT3A-DNMT3L protein with the dCas9-SunTag system and compared its activity with the dCas9-DNMT3A-DNMT3L fusion protein published earlier [19,20]. Both systems demonstrated a strong on-target activity, with dCS being slightly more active (79% versus 84% DNA methylation). However, in this context it needs to be considered that some CpG sites in the analyzed region were already methylated up to 95% by the direct fusion construct, and there was not much dynamic range for further improvement by the SunTag system. Targeted and genome-wide analyses documented strong off-target de novo methylation in CGIs installed by the wild type Ab-3A3L protein, which was introduced due to the untargeted DNA interaction of the DNMT3A-DNMT3L part. This finding is in agreement with a recent study by Galonsaka et al. which showed that off-target methylation is independent of sgRNA or dCas9 and detected mainly in cells expressing the isolated DNMT3A catalytic domain [25].

Recently, two reports demonstrated reduced off-target methylation using the dCas9-SunTag scaffold [24,26] with the DNMT3A catalytic domain as an effector protein in comparison to the dCas9-DNMT3A chimeric protein. There are two main reasons for this effect. The first is that the SunTag approach allows titration of the effector domain and dCas9 individually aiming to achieve high on-target binding on the dCas9 with the optimum concentration of the DNMT3A, which was demonstrated by Pflueger et al. [26]. This is not possible for dCas9-DNMT3A direct fusion since both functional entities are part of a single protein. Secondly, dCas9 and DNMT3A both bind DNA and thus the chimeric proteins might have a higher affinity for DNA than each protein separately. Interaction of dCas9 with sgRNA off-target sites might be stronger in the presence of DNMT3A, and conversely, unspecific DNMT3A-DNA interactions can be facilitated by unspecific dCas9 DNA binding. Both of these effects will lead to higher off-target methylation. The SunTag-based approach, at least in theory, allows dCas9 to find the target DNA sequence and then recruit an effector domain for editing, since the two proteins operate separately until they form a complex. Surprisingly, in our study, the dCS system showed even higher off-target DNA methylation. This disagreement with the previously published results may be explained by variations of experimental conditions such as higher expression levels of the Ab-3A3L protein and its superior enzymatic activity in comparison to Ab-3A used in earlier studies.

As a next step, we aimed to increase the specificity of the dCS by reducing off-target DNA methylation. To achieve this, we designed DNMT3A variants with reduced DNA binding, in order to minimize their unspecific interaction with the DNA. Mutations at three of these residues, R831, K844, and R887, were already tested in vitro and showed weaker DNA binding and strongly reduced enzymatic activity [37,38]. In our targeted methylation experiments in the context of Ab-3A3L, all four DNMT3A variants showed a pronounced increase in specificity, accompanied by a very moderate reduction of on-target activity in comparison to in vitro effect of these mutations reported in the literature. While these results demonstrate the success of the design strategy, they need to be validated for different target regions in future. This contrast of good activity in the target methylation using the dCS system, but low activity in enzymatic essays with purified proteins, can be explained by the targeting and signal amplification effects of the SunTag. A similar approach was utilized by Xiong et al., who demonstrated site-specific DNA methylation in bacteria [41]. In this study, the authors used dCas9 with a split M.SssI methyltransferase and optimized the specificity by targeted mutagenesis of the DNA binding domain of M.SssI. They also were able to reduce off-target methylation significantly.

Additionally, we tried to increase the on-target methylation using a SunTag variant with 24 GCN4 peptide repeats, which surprisingly was not successful and led to even lower on-target methylation. One may speculate that stability and/or folding of the long tag with 24 repeats is lower than that of its shorter version leading to less efficient recruitment of effector domains. More to that, Morita et al. demonstrated that the efficiency of the SunTag could be improved by optimization of the linkers between repeats [29], which might be an approach for further optimization.

In summary, in the current work, we successfully combined a dCas9-SunTag scaffold with a very potent effector protein, DNMT3A-DNMT3L. This combination introduced strong DNA methylation at the *ISG15* target genomic locus in HEK293 cells. We increased the specificity of the system and minimized off-target DNA methylation by designed mutations in the DNMT3A catalytic domain of the DNMT3A-DNMT3L fusion protein. Our data, together with other studies, emphasize that the specificity of the targeted epigenome editing can be improved by the rational design of EpiEditors. Based on our results, the R887E mutant appears particularly promising for future applications as it combines high specificity with high on-target activity.

## 4. Materials and Methods

### 4.1. Cloning

The dC vector was cloned based on the previously published construct [19], in which EYFP was replaced by tagBFP using Gibson Assembly. The tagBFP sequence was obtained from Addgene plasmid #60903. The dCS and dCS24 vectors generated by Tanenbaum et al. [28] (Addgene plasmids #60903 and 60910), were modified by deleting parts of backbone sequences to minimize the size of plasmids. The deletion of unrequired viral elements resulted in a shortening of dCS from 14.5 kb to 10 kb, and dCS24 from 14.9 to 11 kb. To generate the Ab-3A3L vector, a DNMT3A-DNMT3L coding sequence was amplified from dC and sub-cloned into a modified scFV-GCN4-sfGFP-GB1 plasmid (Addgene # 60906) at the BamHI site. scFV-GCN4-sfGFP-GB1 was recloned, which reduced plasmid size from 10 kb to 4.5 kb. sgRNAs were initially cloned into a sgRNA expression vector (Addgene plasmid #41824), then the sgRNA expression cassette was sub-cloned into Addgene plasmid #99914, which provides DsRed expression for FACS. Sequences of all vectors were confirmed by Sanger sequencing. Cloning design and sequence analysis were conducted using the SnapGene software version 3.3.4 (GSL Biotech LLC, Chicago, IL, USA). The sequences of the sgRNAs were GTTCGCTGCCTCTCAGCCGC (ISG15) and GAACAGTCGCGTTTGCGACT (scrambled).

### 4.2. Site-Directed Mutagenesis

All mutations were introduced using a long synthesized primer (megaprimer) with the specific mutation in a rolling circle PCR with the wild type plasmid as a template [42]. Megaprimers were synthesized using a 50 µL reaction mixture containing 1× Pfu buffer, 0.2 mM dNTPs, 0.05 U/μL Pfu polymerase (Thermo Fisher Scientific, Waltham, MA, USA), 0.4 μM forward and 0.4 μM reverse primers (containing the mutation), and 5 ng of plasmid DNA with the following cycle conditions: 3 min at 95 °C, 40 cycles of 30 s at 94 °C, 30 s at 58 °C, 1 min at 72 °C, and finally 5 min at 72 °C. The PCR products were verified by agarose gel electrophoresis and purified with the NucleoSpin Gel and PCR Clean-up Kit (Macherey-Nagel, Düren, Germany). The purified megaprimers were used for rolling circle PCR with the following cycle conditions: 3 min at 95 °C, 35 cycles of 30 s at 94 °C, 30 s at 72 °C, 29 min at 72 °C, and finally 40 min at 72 °C; and a reaction mixture containing 1× Pfu buffer, 0.2 mM dNTPs, 0.05 U/μL Pfu polymerase (Thermo Fisher Scientific), 250 ng synthesized megaprimer, and 50 ng of plasmid DNA. DpnI digest was performed at 37 °C after adding 5.6 µL Cutsmart and 1 µL enzyme to the PCR products to remove the original DNA template. After DpnI deactivation at 80 °C for 20 min, the digested PCR products were purified with the NucleoSpin Gel and PCR Clean-up Kit, eluted with 15 µL water and 5 µL were used for transformation into electrocompetent XL1 blue cells. Plasmids were purified using the Plasmid DNA Purification Kit (Macherey-Nagel). All constructs were verified by Sanger sequencing.

### 4.3. Cell Culture Co-Transfections and FACS

HEK293 cells were cultivated at 37 °C in a cell culture incubator with 95% relative humidity and 5% (*v*/*v*) CO_2_. Cells were cultured in DMEM (Dulbecco’s modified Eagle’s medium, Sigma-Aldrich, St. Louis, MO, USA) supplemented with 10% (*v*/*v*) fetal bovine serum (FBS, Sigma-Aldrich), 2% (*v*/*v*) L-glutamine (Sigma-Aldrich), and 1% (*v*/*v*) Penicillin/Streptomycin (Sigma-Aldrich).

Transfections of HEK293 cells were carried out at 70% confluency 24 h post-seeding in a 10 cm petri dish using FuGENE HD (Promega, Fitchburg, WI, USA) as transfection reagent following the manufacturer’s instructions. For transfections of the SunTag system, 6 µg of dCS construct, 3 µg of the Ab-3A3L construct, and 500 ng of the sgRNA coding vectors were pooled. The dCas9-3A3L direct fusion system was transfected as a mixture of 6 µg of dC construct, 500 ng of the sgRNA coding vectors, and 3 µg of empty plasmid to keep the total amount of DNA at 9.5 µg as used for the SunTag system transfections. Cells were harvested by trypsinization 72 h later and filtered through a 30 µm pre-separation filter (Miltenyi Biotec, Bergisch Gladbach, Germany) followed by preparative cell sorting by flow cytometry. In the case of the SunTag system, triple-positive cells expressing tagBFP (dCS), GFP (Ab-3A3L), and DsRed (ISG15 gRNA) were sorted using a Sony Biotechnology (San Jose, CA, USA) CellSorter SH800S. For the direct fusion system, double-positive cells expressing the reporter genes tagBFP (dC) and DsRed (ISG15 gRNA) were sorted. For all channels, compensation from single transfections was applied to avoid spillover. In general, 0.5–1 × 10^6^ cells were collected, pelleted at 200× *g* for 5 min and treated subsequently for the extraction of genomic DNA (gDNA).

### 4.4. Targeted DNA Methylation Analysis

Methylation of 19 CpG sites at the *ISG15* promoter region (chr1:948633-948924, hg19) and 12 CpG sites at the *VEGFA* promoter region (chr6:43738171-43738372, hg19) were analyzed in one DNA strand using targeted DNA methylation. Bisulfite conversion of 500 ng gDNA was performed using the EZ DNA Methylation-Lightning kit (Zymo Research, Irvine, CA, USA) according to the manufacturer’s protocol. Afterward, a two-step PCR approach was used to generate DNA libraries, which were sent for Illumina Next Generation Sequencing (NGS). Therefore, in the first PCR (PCR1), 1 µL of bisulfite converted and purified DNA was amplified with primers containing internal barcodes (Table S2) using a mixture of 1× PCR Buffer, 0.2 mM dNTPs, 0.05 U/μL HotStartTaq DNA Polymerase (QIAGEN, Venlo, Netherlands), and 0.5 μM forward and 0.5 μM reverse primers in a total volume of 20 µL and the following PCR conditions: 15 min at 95 °C, 15 cycles of 30 s at 94 °C, 30 s at 50 °C, 1 min at 72 °C, and finally 5 min at 72 °C. The obtained PCR1 products were diluted 2–10-fold and 1 µL of this dilution was used as a template for a second PCR (PCR2) with another set of primers (Table S2) to introduce adapters and indices needed for NGS. For this amplification the following reaction conditions were used: 30 s at 98 °C, 15 cycles of 10 s at 98 °C, 40 s at 72 °C, and 5 min at 72 °C; using a mixture containing 1× Phusion HF Buffer, 0.2 mM dNTPs, 0.02 U/μL Phusion HF DNA Polymerase (Thermo Fis-her Scientific), and 0.4 μM forward and 0.4 μM reverse primers in a total volume of 20 μL. Purification was performed for a pool of DNA libraries including equimolar amounts of each sample using NucleoSpin^®^ Gel and PCR Clean-up kit (Macherey-Nagel).

Sequencing was performed on Illumina MiSeq using paired-end 2 × 250 cycles protocol. At least 3000 reads were obtained for every amplicon and sample. NGS data were analyzed as described earlier [43] on a local Galaxy server [44]. Adapters and low-quality ends (score below 20) were trimmed by Trim Galore! tool (developed by Felix Krueger at the Babraham Institute) and merged using Pear [45] with a minimum overlap size set to 20. The reads were aligned to the reference sequence using the bwameth tool (Brent, 2014, arXiv:1401.1129v2) and DNA methylation levels for individual CpG sites were extracted using MethylDackel (https://github.com/dpryan79/MethylDackel, developed by Devon Ryan). The following analysis was done using Microsoft Excel 2016 (Redmond, WA, USA).

### 4.5. Genome-Wide DNA Methylation Analysis by MBD2-Pulldown Coupled with NGS

Genomic DNA (gDNA) from 0.5–1 × 10^6^ cells was isolated using the QIAamp DNA Mini Kit (QIAGEN) and eluted with 200 µL ddH_2_O pre-warmed to 70 °C. Fragmentation was performed by sonication using the EpiShear™ probe sonicator (Active Motif, Carlsbad, CA; USA) with the 2 mm tip (settings: 25% amplitude, 20 × 20 s pulse/30 s pause). The fragmented gDNA was re-purified and concentrated using the NucleoSpin Gel and PCR Clean-up Kit (Macherey-Nagel). In pulldown experiments, 1 µg of fragmented gDNA was mixed with 8.75 µg GST tagged MBD2 in a final volume of 250 µL ice-cold PB150 buffer (50 mM Tris-HCl pH8.0, 150 mM NaCl, 1 mM EDTA, 0.5% Igepal CA-630, 2 mM DTT). Samples were incubated overnight under constant rotation at 4 °C. The next day, 50 µL of glutathione agarose beads (Macherey-Nagel) were washed four times with ice-cold PB150 and pulldown samples were transferred to the beads. Immobilization of GST-MBD2 to beads was performed under constant rotation at 4 °C for 2 h. Afterward, beads were washed three times with 200 µL ice-cold PB500 buffer (50 mM Tris-HCl pH8.0, 500 mM NaCl, 1 mM EDTA, 0.5% Igepal CA-630, 2 mM DTT) for 5 min under constant rotation at 4 °C and after the final wash, the supernatant was removed. For elution, 150 µL PB2000 buffer (10 mM Tris-HCl pH8.0, 2 M NaCl, 1 mM EDTA) was added and samples were rotated for 15 min at RT. The supernatant was transferred into a reaction tube and the elution was repeated with an additional 150 µL of PB2000. After combining both elution fractions, the samples were purified using the ChIP DNA Purification Kit (Active Motif). The precipitated DNA was finally eluted in 50 µL elution buffer provided in the kit.

Library preparation was performed using the NEBNext^®^ Ultra™ II DNA Library Prep Kit for Illumina^®^ (New England Biolabs, Ipswich, MA, USA) and barcodes for each sample were taken from NEBNext^®^ Multiplex Oligos for Illumina^®^ (Index Primers Set 1/Dual Index Primers Set 1) (New England Biolabs). Therefore, 15 µL from samples obtained by MBD2-pulldown were used and treated as described in the manufacturer’s protocol. The desired size distribution of the libraries was confirmed by LabChip^®^ GXII Touch™ HT system (Perkin Elmer, Waltham, MA, USA) and 600 ng of the barcoded samples were pooled. Next-Generation Sequencing was performed by the Max Planck-Genome-Centre Cologne utilizing the Illumina^®^ HiSeq3000 platform with, on average, 10 million reads per sample (150 bp single-end reads).

Sequencing files were obtained in FASTQ format and all data processing steps were performed on the European Galaxy web platform (https://usegalaxy.eu/) [44]. The quality of the reads was analyzed via the control tool FastQC (developed by Simon Andrews at the Babraham Institute) and reads were mapped on the human genome hg19 using the alignment tool Bowtie2 [46] under default settings. Coverage bigwig files were obtained with 10 bp bin size resolution and data were normalized to RPKM using the bamCoverage tool [47]. An internal re-calibration was applied, because RPKM normalization alone is incorrect if there is a large increase in peak number.

For the internal re-normalization, coordinates of the CGIs were taken from the UCSC Table Browser (https://genome.ucsc.edu/index.html) and signals of bigwig-files in CGIs were quantified with the multiBigwigSummary [47] tool. Signals of the “untreated” sample were sorted in descending order, the 1000 most highly methylated CGIs were selected, and the intensities of all data sets were normalized to the average intensities in these regions. The rationale for using these regions was that they were already fully methylated before DNMT treatment and, hence, DNA methylation was not expected to increase. New coverage bigwig files were prepared using the same settings but considering the calculated scaling factor. Heatmaps were created using the tools computeMatrix [47] and plotHeatmap [47]. Browser views of MBD-seq data were created with the Integrative Genomics Viewer (https://software.broadinstitute.org/software/igv/). The data are available at GEO under the accession number: GSE142181.

## Supplementary Materials

Supplementary materials can be found at https://www.mdpi.com/1422-0067/21/2/502/s1.
