# Supplementary material for: Engineering of Effector Domains for Targeted DNA Methylation with Reduced Off-Target Effects
Source: Int J Mol Sci. 2020 Jan 13;21(2):502. doi: 10.3390/ijms21020502 (PMC7013458; doi:10.3390/ijms21020502)
Supplement: Supplementary file 1 [file ijms-21-00502-s001.pdf]

Engineering of effector domains for targeted DNA methylation with reduced off-target effects

Daniel Hofacker[1], Julian Broche[1], Laura Laistner, Sabrina Adam, Pavel Bashtrykov* and Albert Jeltsch*

[1]These authors contributed equally to this work.

*Corresponding authors.

Supplemental materials

Supplementary figures

Supplementary figure 1: Compilation of bisulfite conversion rates.

Supplemental figure 2: Compilation of the bis-seq DNA methylation data shown in aggregated form in Figure 2-4.

Supplementary figure 3: New DNA methylation peaks identified after treatment of cells with dCS, Ab-3A3L and the ISG15 sgRNA.

Supplementary figure 4: Signals of MBD-seq data in the CGIs of the *ISG15*, *VEGFA*, *ACTB* and *GAPDH* genes extracted using the multiBigwigSummary tool.

Supplementary figure 5: IGV views of MBD-seq data displayed for the two additional off-target regions *ACTB* and *GAPDH*.

Supplementary tables

Supplementary table 1: Potential off-target binding sites of the *ISG15* sgRNA used in this study as identified by http://www.rgenome.net/cas-offinder/.

Supplementary table 2: Primers used in this work.

Supplementary figure 1: Compilation of bisulfite conversion rates. Bisulfite conversion was determined based on the read count of cytosine residues at non-CpG sites after bisulfite conversion of genomic DNA isolated from untransfected cells in two experimental repeats (Rep. 1 and Rep. 2).

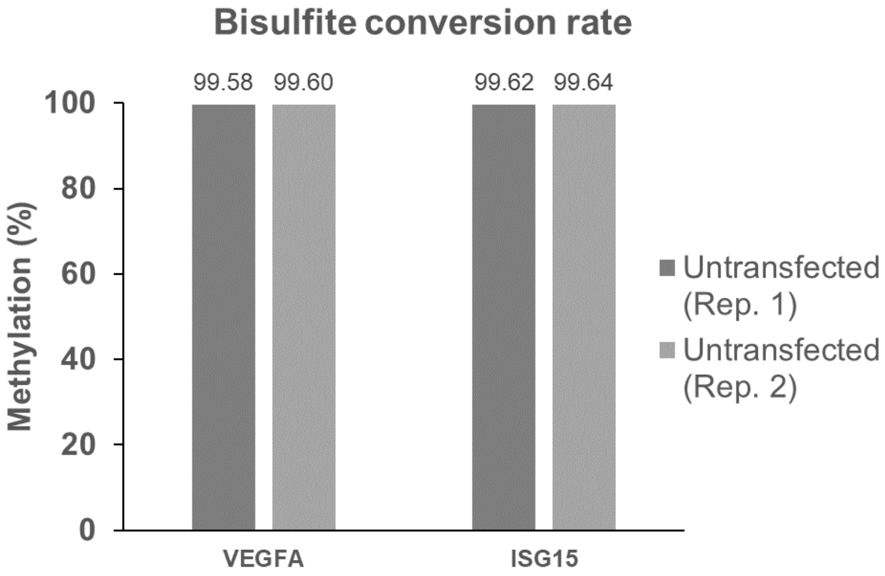

Supplemental figure 2: Compilation of the bis-seq DNA methylation data shown in aggregated form in Figure 2-4. In each case, the x-axis displays the individual numbered CpG sites. For the experimental conditions, refer to the legends of the individual main figures.

Methylation of VEGFA in Fig. 2D

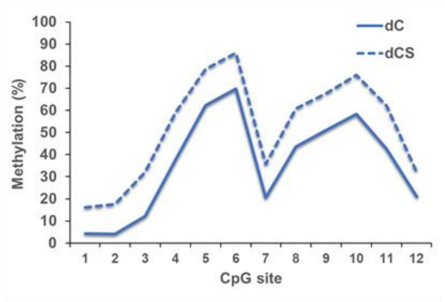

Methylation of ISG15 and VEGFA in Fig. 3B

ISG15

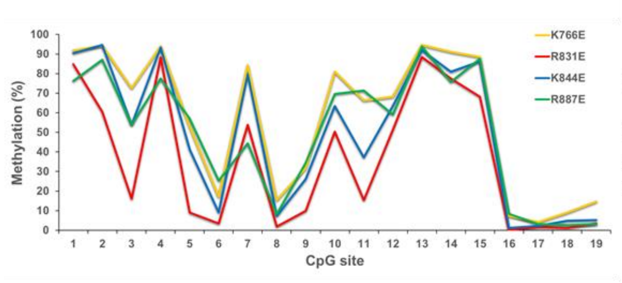

VEGFA

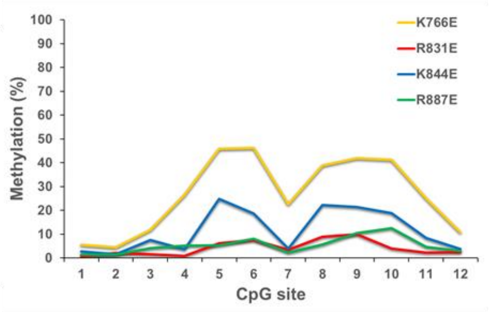

Methylation of VEGFA in Fig. 3D

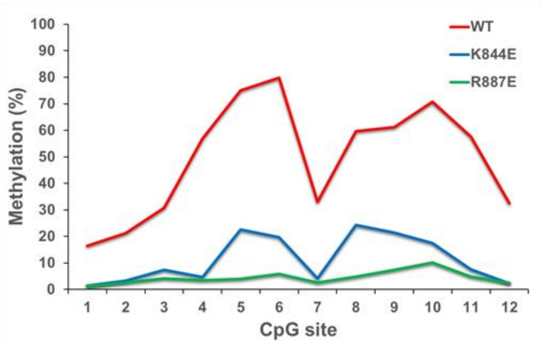

Methylation of VEGFA in Fig. 5C

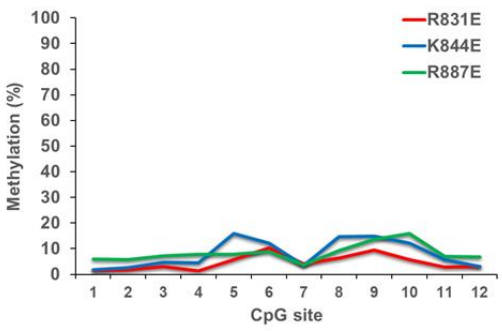

Supplementary figure 3: New DNA methylation peaks identified after treatment of cells with dCS, Ab-3A3L and the ISG15 sgRNA. Newly appearing MBD-seq peaks were intersected with all CGIs. 1943 out of 12,864 newly appearing MBD-seq peaks (15%) were not within an annotated CGI. Among them, only 263 peaks were more than 1 kb apart from the next CGI (2% of all newly appearing peaks).

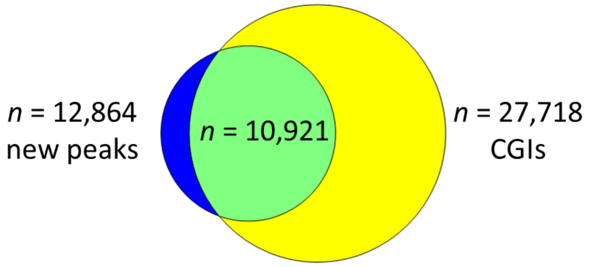

Supplementary figure 4: Signals of MBD-seq data in the CGIs of the *ISG15*, *VEGFA*, *ACTB* and *GAPDH* genes extracted using the multiBigwigSummary tool. Note the reduced relative activity of the K844E and R887E variants at the off-target sites. Cells were treated with dCS, Ab-3A3L with indicated mutations in the DNMT3A part and the ISG15 sgRNA.

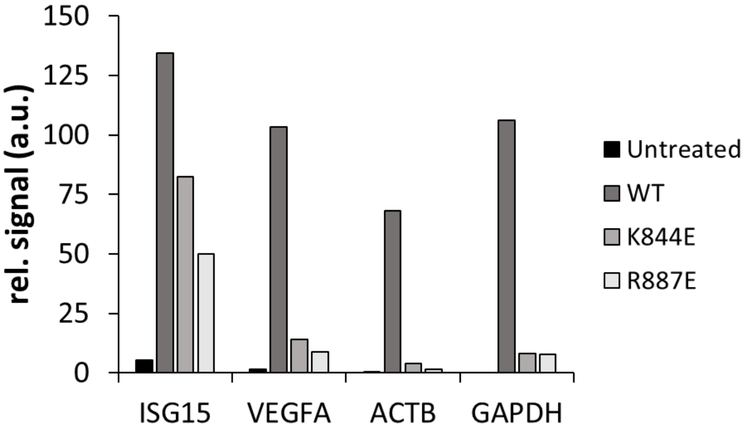

Supplementary figure 5: IGV views of MBD-seq data displayed for the two additional off-target regions *ACTB* and *GAPDH*. Cells were treated with dCS, Ab-3A3L with indicated mutations in the DNMT3A part and the ISG15 sgRNA. The wild type DNMT3A (WT) demonstrated higher off-target methylation levels than the K844E or R887E variants.

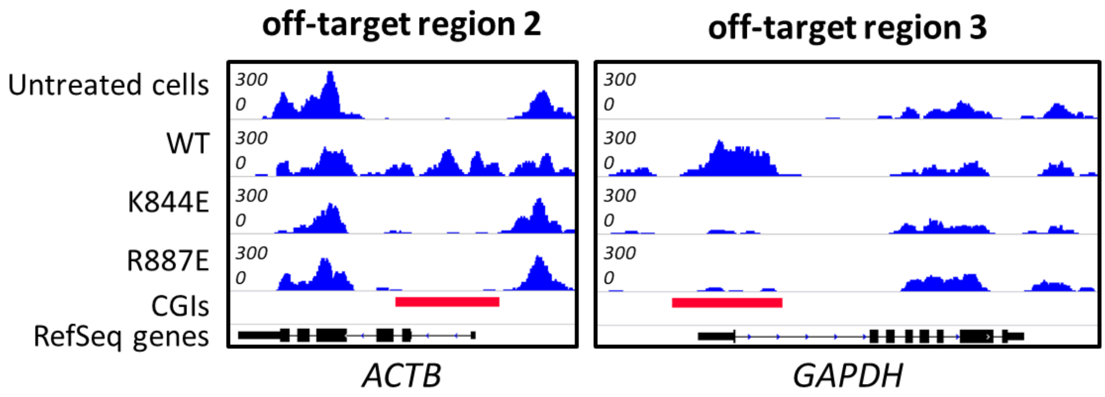

Supplementary table 1: Potential off-target binding sites of the *ISG15* sgRNA used in this study as identified by http://www.rgenome.net/cas-offinder/.

#Bulge type	crRNA	DNA	Chromo-some	Position	Direc-tion	Mis-matches	Bulge Size
X	GTTCGCTGCCTCTCAGCC GCNGG	GgTCGCTGCCTCTCcaCCG gAGG	chr8	17104884	+	4	0
X	GTTCGCTGCCTCTCAGCC GCNGG	GTTaGCTGCCTCTCgGtCG CCGG	chr8	43406533	-	3	0
X	GTTCGCTGCCTCTCAGCC GCNGG	GaTCGaTtCCTCTCAGCCa CAGG	chr8	62815042	-	4	0
X	GTTCGCTGCCTCTCAGCC GCNGG	aTTCaCaGCaTCTCAGCCG CAGG	chr8	81805938	+	4	0
X	GTTCGCTGCCTCTCAGCC GCNGG	tTTCcCTtCCTCTCAGCCcC TGG	chr8	124933078	+	4	0
X	GTTCGCTGCCTCTCAGCC GCNGG	tTTtGCTGCCTgTCAGCtGC AGG	chr12	73776344	+	4	0
X	GTTCGCTGCCTCTCAGCC GCNGG	GTTCGtTaaCTCTCAGCtG CAGG	chr12	86975240	-	4	0
X	GTTCGCTGCCTCTCAGCC GCNGG	GgaaGCTGCCaCTCAGCC GCAGG	chr12	95397507	+	4	0
X	GTTCGCTGCCTCTCAGCC GCNGG	tTTCtCTGCCTCTCAGCtGg AGG	chr12	105098325	+	4	0
X	GTTCGCTGCCTCTCAGCC GCNGG	GTTCcCTcCCTCTCAcCtGC TGG	chr12	112605777	+	4	0
X	GTTCGCTGCCTCTCAGCC GCNGG	tTTCtCTGCCaCTCAGaCG CAGG	chr12	124094533	+	4	0
X	GTTCGCTGCCTCTCAGCC GCNGG	aTTCcCTGCCTtTCAGCCG gTGG	chr3	51727405	+	4	0
X	GTTCGCTGCCTCTCAGCC GCNGG	GTTCtCTaCCTCcCAGCCtC TGG	chr3	59181111	-	4	0
X	GTTCGCTGCCTCTCAGCC GCNGG	GTTaGCTGgCTgTCAGCgG CAGG	chr3	132824091	+	4	0
X	GTTCGCTGCCTCTCAGCC GCNGG	GTTaGCTGtCTCTtgGCCG CCGG	chr3	186728772	+	4	0
X	GTTCGCTGCCTCTCAGCC GCNGG	GTTCtCctCCTCTCAGCCcC TGG	chr7	2508465	+	4	0
X	GTTCGCTGCCTCTCAGCC GCNGG	GgTtGCTGCCTCTCAGCCc tAGG	chr7	50567760	+	4	0
X	GTTCGCTGCCTCTCAGCC GCNGG	GTaCGCTGCCTCcCcGCCG CCGG	chr7	101460483	+	3	0
X	GTTCGCTGCCTCTCAGCC GCNGG	GTTCcCTGgCcCTgAGCCG CGGG	chr7	139344168	+	4	0
X	GTTCGCTGCCTCTCAGCC GCNGG	GTTaGCTGtCTCTtgGCCG CTGG	chr4	8430973	-	4	0
X	GTTCGCTGCCTCTCAGCC GCNGG	GTTaGCTGCCTCTCgGtCG CCGG	chr4	25522958	+	3	0
X	GTTCGCTGCCTCTCAGCC GCNGG	GTTCcCgGCtTCTCAGCCa CTGG	chr4	40337256	+	4	0
X	GTTCGCTGCCTCTCAGCC GCNGG	GTTCtCTGCCTCTCtGCCtg AGG	chr4	75658461	+	4	0
X	GTTCGCTGCCTCTCAGCC GCNGG	tTcCGCTGaCTCTCAGCCc CGGG	chr4	109572018	+	4	0
X	GTTCGCTGCCTCTCAGCC GCNGG	GTTaGCTGCacCTCAGCtG CAGG	chr4	141746720	+	4	0
X	GTTCGCTGCCTCTCAGCC GCNGG	GgcCGCaGCCTCTCAGCC GCCGG	chr5	14664836	-	3	0

X	GTTCGCTGCCTCTCAGCC GCNGG	GTTCGCTGCCTgaCAtCtG CAGG	chr16	4884173	-	4	0
X	GTTCGCTGCCTCTCAGCC GCNGG	GTgCGCTGCCTCcCAGgaG CCGG	chr16	12213529	+	4	0
X	GTTCGCTGCCTCTCAGCC GCNGG	GaTCGCTGCtTCcCAGCCt CAGG	chr16	27250582	+	4	0
X	GTTCGCTGCCTCTCAGCC GCNGG	GTTCGCTGCCTCTCAGCC GCCGG	chr1	948874	-	0	0
X	GTTCGCTGCCTCTCAGCC GCNGG	tTTCcCTGtCTCTCAGCtGC AGG	chr1	17986443	-	4	0
X	GTTCGCTGCCTCTCAGCC GCNGG	GTgCcCTcCtTCTCAGCCG CTGG	chr1	26508857	-	4	0
X	GTTCGCTGCCTCTCAGCC GCNGG	GacCcCTGCCTCTCAGCCc CTGG	chr1	32716729	-	4	0
X	GTTCGCTGCCTCTCAGCC GCNGG	GTTCcCTGaCTCTCAcCCtC GGG	chr1	41760952	-	4	0
X	GTTCGCTGCCTCTCAGCC GCNGG	GTTaGCcatCTCTCAGCCG CTGG	chr1	146710912	+	4	0
X	GTTCGCTGCCTCTCAGCC GCNGG	GTTCaCTGCCTCTCAGCtG tGGG	chr1	215131205	+	3	0
X	GTTCGCTGCCTCTCAGCC GCNGG	GgTCctTGCCTCTCAGCtG CTGG	chr1	220325057	-	4	0
X	GTTCGCTGCCTCTCAGCC GCNGG	tTcCtCTGCCTCTCAGCCtC CGG	chr1	225931264	-	4	0
X	GTTCGCTGCCTCTCAGCC GCNGG	GTgCGCTtCCTCTgAGCCa CTGG	chr13	26423100	+	4	0
X	GTTCGCTGCCTCTCAGCC GCNGG	GcTgGCcGCCTCTCcGCCG CTGG	chr2	90284246	+	4	0
X	GTTCGCTGCCTCTCAGCC GCNGG	GcTgGCcGCCTCTCcGCCG CTGG	chr2	90284228	+	4	0
X	GTTCGCTGCCTCTCAGCC GCNGG	GcTgGCcGCCTCTCcGCCG CTGG	chr2	90527824	-	4	0
X	GTTCGCTGCCTCTCAGCC GCNGG	GcTgGCcGCCTCTCcGCCG CTGG	chr2	90527806	-	4	0
X	GTTCGCTGCCTCTCAGCC GCNGG	GgTgGCcGCCTCTCcGCCG CTGG	chr2	91750692	-	4	0
X	GTTCGCTGCCTCTCAGCC GCNGG	GTTCcCTGCCTCcCAGCCc aGGG	chr2	96763987	+	4	0
X	GTTCGCTGCCTCTCAGCC GCNGG	GTTacCTGCCTCTCAGCCc tGGG	chr2	161960608	+	4	0
X	GTTCGCTGCCTCTCAGCC GCNGG	GTgCGCTGCCTCTgccCCG CGGG	chr2	175205238	+	4	0
X	GTTCGCTGCCTCTCAGCC GCNGG	GTTCcCTGCaTCTCAtCCcC AGG	chr2	199599312	-	4	0
X	GTTCGCTGCCTCTCAGCC GCNGG	GTTaGCTGtCTCTtgGCCG CCGG	chr2	201990826	-	4	0
X	GTTCGCTGCCTCTCAGCC GCNGG	GcTgGCTGCtTCTCAGCaG CTGG	chr22	27533888	+	4	0
X	GTTCGCTGCCTCTCAGCC GCNGG	aTTaGCTGCCTCTCgGtCG CCGG	chr22	39507745	+	4	0
X	GTTCGCTGCCTCTCAGCC GCNGG	GTTtcCcGCCTCTCAGgCG CGGG	chr22	41864477	-	4	0
X	GTTCGCTGCCTCTCAGCC GCNGG	tcTCGCTGCgcCTCAGCCG CAGG	chr19	2207686	+	4	0
X	GTTCGCTGCCTCTCAGCC GCNGG	GTTCctTcCCTCTCAGCCtC TGG	chr19	8828214	-	4	0

X	GTTCGCTGCCTCTCAGCCGCNGG	GTTaGCTGtCTCTtgGCCGCTGG	chr19	23369142	-	4	0
X	GTTCGCTGCCTCTCAGCCGCNGG	GgTCGCTGaCTCaCAGCCGCAGG	chr19	46010136	-	3	0
X	GTTCGCTGCCTCTCAGCCGCNGG	GcTaGCTGtCTCTCAGtCGCTGG	chr19	51253303	+	4	0
X	GTTCGCTGCCTCTCAGCCGCNGG	tgTCtCTGCCTCTCAGCCcCCGG	chr19	51585940	-	4	0
X	GTTCGCTGCCTCTCAGCCGCNGG	aTggGCTGCCaCTCAGCCGCGGG	chr21	44775160	+	4	0
X	GTTCGCTGCCTCTCAGCCGCNGG	GTTaGCTGgCTCTCAGCCtCAGG	chr15	28106352	+	3	0
X	GTTCGCTGCCTCTCAGCCGCNGG	GTTaGCTtCCTtTCAGCCtCTGG	chr15	94806594	+	4	0
X	GTTCGCTGCCTCTCAGCCGCNGG	GTTaGCTGCCTCTCcGtCGCTGG	chrY	9938947	+	3	0
X	GTTCGCTGCCTCTCAGCCGCNGG	GTTtGgTGCCTgTCAGCCGtGGG	chr17	4982094	+	4	0
X	GTTCGCTGCCTCTCAGCCGCNGG	GcTtGCTGCCTtTCAGCaGCTGG	chr17	17143558	-	4	0
X	GTTCGCTGCCTCTCAGCCGCNGG	GTcCtCTGCCTaTCAGaCGCCGG	chr17	21187912	-	4	0
X	GTTCGCTGCCTCTCAGCCGCNGG	GcTaGCTGtCTCTCAGtCGCCGG	chr17	66065887	-	4	0
X	GTTCGCTGCCTCTCAGCCGCNGG	GgTCctTGCCTCgCAGCCGCCGG	chr6	5260751	+	4	0
X	GTTCGCTGCCTCTCAGCCGCNGG	GTgCGCctCCTCTCAGCaGCCGG	chr6	40638930	+	4	0
X	GTTCGCTGCCTCTCAGCCGCNGG	GTgCGCTGggTCTCAGCCcCGGG	chr6	111303336	+	4	0
X	GTTCGCTGCCTCTCAGCCGCNGG	GTTCtCTGCCTgTgAGCCcCTGG	chr6	145779528	-	4	0
X	GTTCGCTGCCTCTCAGCCGCNGG	GTTaGCcGtCTCTCAGtCGCCGG	chr6	153003705	+	4	0
X	GTTCGCTGCCTCTCAGCCGCNGG	GTTCcCTGCCTCTtAGCtGgAGG	chr14	24576358	-	4	0
X	GTTCGCTGCCTCTCAGCCGCNGG	GTTCaCTGCCTCTgAGCtGgTGG	chr14	47936202	-	4	0
X	GTTCGCTGCCTCTCAGCCGCNGG	GTTgGgTGCCTCTCAGCCGtGGG	chr14	56824336	+	3	0
X	GTTCGCTGCCTCTCAGCCGCNGG	GTTCGCTGCgcCgCgGCCGCCGG	chr14	75745570	-	4	0
X	GTTCGCTGCCTCTCAGCCGCNGG	cTTCcCTaCCTgTCAGCCGCTGG	chr14	78346500	+	4	0
X	GTTCGCTGCCTCTCAGCCGCNGG	GTTaGCTGtCTCTtgGCCGCCGG	chr14	101561691	+	4	0
X	GTTCGCTGCCTCTCAGCCGCNGG	GTTCaCTGCCTCTCtcCCcCAGG	chr20	15601656	-	4	0
X	GTTCGCTGCCTCTCAGCCGCNGG	cTTgGCTcCCTCTCAGCtGCTGG	chr9	87015605	-	4	0
X	GTTCGCTGCCTCTCAGCCGCNGG	GcTgGCTGCCTCTCAGCaGtGGG	chr9	95402183	-	4	0
X	GTTCGCTGCCTCTCAGCCGCNGG	tcTCtCTGaCTCTCAGCCGCTGG	chr9	128801012	-	4	0
X	GTTCGCTGCCTCTCAGCCGCNGG	ccTCcCTGCCTCTCAGCCcCTGG	chr9	132005010	-	4	0

X	GTTCGCTGCCTCTCAGCCGCNGG	GTTCtCTGCCTCaCAGgaGCCGG	chr9	138022430	+	4	0
X	GTTCGCTGCCTCTCAGCCGCNGG	cTTtGCTtCCTCTCAGCaGCCGG	chr9	137943631	-	4	0
X	GTTCGCTGCCTCTCAGCCGCNGG	GTTCaCTGCCaCTCAGCCtgAGG	chrX	44601032	+	4	0
X	GTTCGCTGCCTCTCAGCCGCNGG	GTTCcCTGCCaaTCAGCCtCTGG	chrX	53032189	+	4	0
X	GTTCGCTGCCTCTCAGCCGCNGG	GTTaGCTGCCTCTCgGtCGCCGG	chr18	52057540	-	3	0
X	GTTCGCTGCCTCTCAGCCGCNGG	GTTgGCaGCaTCTCAGCCGCAGG	chr18	56452683	-	3	0
X	GTTCGCTGCCTCTCAGCCGCNGG	GTTCcCTGCaTCTCAaCCaCAGG	chr11	6072400	-	4	0
X	GTTCGCTGCCTCTCAGCCGCNGG	GTTCGCTGCCTCaCAGttcCTGG	chr11	18765551	-	4	0
X	GTTCGCTGCCTCTCAGCCGCNGG	GcTCtCTGCCTCTgAGCCtCAGG	chr11	48542507	+	4	0
X	GTTCGCTGCCTCTCAGCCGCNGG	GcTCtCTGCCTCTgAGCCtCAGG	chr11	48643596	+	4	0
X	GTTCGCTGCCTCTCAGCCGCNGG	GaTCtCTGCCTCTgAGCCtCAGG	chr11	48607292	+	4	0
X	GTTCGCTGCCTCTCAGCCGCNGG	GcTCtCTGCCTCTgAGCCtCAGG	chr11	51400389	-	4	0
X	GTTCGCTGCCTCTCAGCCGCNGG	GcTCtCTGCCTCTgAGCCtCGGG	chr11	51431909	-	4	0
X	GTTCGCTGCCTCTCAGCCGCNGG	GcTCtCTGCCTCTgAGCCtCAGG	chr11	55081356	+	4	0
X	GTTCGCTGCCTCTCAGCCGCNGG	GcTCtCTGCCTCTgAGCCtCAGG	chr11	55151318	+	4	0
X	GTTCGCTGCCTCTCAGCCGCNGG	GTTCtCTGCCTCTgAGCCtCAGG	chr11	55203729	+	3	0
X	GTTCGCTGCCTCTCAGCCGCNGG	GaTCtCTGCCTCTgAGCCtCAGG	chr11	55238900	+	4	0
X	GTTCGCTGCCTCTCAGCCGCNGG	GcTCtCTGCCTCTgAGCCtCAGG	chr11	55193084	+	4	0
X	GTTCGCTGCCTCTCAGCCGCNGG	tTTaGCTGCCTCTCAaCtGCAGG	chr11	64007861	+	4	0
X	GTTCGCTGCCTCTCAGCCGCNGG	GccCaCaGCCTCTCAGCCGCAGG	chr11	69354395	+	4	0
X	GTTCGCTGCCTCTCAGCCGCNGG	GTTCcaTGCCTCTCAGCttCTGG	chr11	73316844	-	4	0
X	GTTCGCTGCCTCTCAGCCGCNGG	GagCcCTGCCTCTCAGCCtCGGG	chr11	76886328	+	4	0
X	GTTCGCTGCCTCTCAGCCGCNGG	GgTgGCTGaCTCTCAGCaGCCGG	chr11	117917816	-	4	0
X	GTTCGCTGCCTCTCAGCCGCNGG	GTTaGCcGCCTCTCgGgCGCTGG	chr11	125555674	+	4	0

Supplementary table 2: Primers used in this work.

Nucleotides highlighted by red color are inline barcodes, nucleotides printed in green are indices. NNNNN – random nucleotides, which were introduced to increase the sequence diversity of the library.

	ISG15 primers for bis PCR1
FP1	ACACTCTTTCCCTACACGACGCTCTTCCGATCTNNNNN**AGAGC**TTAGGTGTTTTTAGGGTGTTGG
RP1	GTGACTGGAGTTCAGACGTGTGCTCTTCCGATCTNNNNN**AGCAT**CACAAACTCCTATACTAACAAAAATAAAT
FP2	ACACTCTTTCCCTACACGACGCTCTTCCGATCTNNNNN**GACAT**TTAGGTGTTTTTAGGGTGTTGG
RP2	GTGACTGGAGTTCAGACGTGTGCTCTTCCGATCTNNNNN**GTCGT**CACAAACTCCTATACTAACAAAAATAAAT
FP3	ACACTCTTTCCCTACACGACGCTCTTCCGATCTNNNNN**ATAGA**TTAGGTGTTTTTAGGGTGTTGG
RP3	GTGACTGGAGTTCAGACGTGTGCTCTTCCGATCTNNNNN**AAGCG**CACAAACTCCTATACTAACAAAAATAAAT
FP4	ACACTCTTTCCCTACACGACGCTCTTCCGATCTNNNNN**GTGTA**TTAGGTGTTTTTAGGGTGTTGG
RP4	GTGACTGGAGTTCAGACGTGTGCTCTTCCGATCTNNNNN**GTCAC**CACAAACTCCTATACTAACAAAAATAAAT

	VEGFA primers for bis PCR1
FP1	GTGACTGGAGTTCAGACGTGTGCTCTTCCGATCTNNNNN**AGAGC**GTTTGTTATTTTTTATTTGAAT
RP1	ACACTCTTTCCCTACACGACGCTCTTCCGATCTNNNNN**AGCAT**AATCACTCACTTTACCCCTATC
FP2	GTGACTGGAGTTCAGACGTGTGCTCTTCCGATCTNNNNN**TGTCG**GTTTGTTATTTTTTATTTGAAT
RP2	ACACTCTTTCCCTACACGACGCTCTTCCGATCTNNNNN**TCAGC**AATCACTCACTTTACCCCTATC
FP3	GTGACTGGAGTTCAGACGTGTGCTCTTCCGATCTNNNNN**GACAT**GTTTGTTATTTTTTATTTGAAT
RP3	ACACTCTTTCCCTACACGACGCTCTTCCGATCTNNNNN**GTCGT**AATCACTCACTTTACCCCTATC
FP4	GTGACTGGAGTTCAGACGTGTGCTCTTCCGATCTNNNNN**CAGAT**GTTTGTTATTTTTTATTTGAAT
RP4	ACACTCTTTCCCTACACGACGCTCTTCCGATCTNNNNN**CGTGT**AATCACTCACTTTACCCCTATC

	PCR2 primers with indices and Illumina adapters
P5 primers	AATGATACGGCGACCACCGAGATCTACAC**ATTACTCG**ACACTCTTTCCCTACACGACGCTCTTCCGATCT
	AATGATACGGCGACCACCGAGATCTACAC**TCCGGAGA**ACACTCTTTCCCTACACGACGCTCTTCCGATCT
	AATGATACGGCGACCACCGAGATCTACAC**CGCTCATT**ACACTCTTTCCCTACACGACGCTCTTCCGATCT
	AATGATACGGCGACCACCGAGATCTACAC**GAGATTCC**ACACTCTTTCCCTACACGACGCTCTTCCGATCT
	AATGATACGGCGACCACCGAGATCTACAC**ATTCAGAA**ACACTCTTTCCCTACACGACGCTCTTCCGATCT
	AATGATACGGCGACCACCGAGATCTACAC**GAATTCGT**ACACTCTTTCCCTACACGACGCTCTTCCGATCT
P7 primers	CAAGCAGAAGACGGCATACGAGAT**CGAGTAAT**GTGACTGGAGTTCAGACGTGTGCTCTTCCGATCT
	CAAGCAGAAGACGGCATACGAGAT**TCTCCGGA**GTGACTGGAGTTCAGACGTGTGCTCTTCCGATCT
	CAAGCAGAAGACGGCATACGAGAT**AATGAGCG**GTGACTGGAGTTCAGACGTGTGCTCTTCCGATCT
	CAAGCAGAAGACGGCATACGAGAT**GGAATCTC**GTGACTGGAGTTCAGACGTGTGCTCTTCCGATCT
	CAAGCAGAAGACGGCATACGAGAT**TTCTGAAT**GTGACTGGAGTTCAGACGTGTGCTCTTCCGATCT
	CAAGCAGAAGACGGCATACGAGAT**ACGAATTC**GTGACTGGAGTTCAGACGTGTGCTCTTCCGATCT
